# The Use of Menthol in Skin Wound Healing—Anti-Inflammatory Potential, Antioxidant Defense System Stimulation and Increased Epithelialization

**DOI:** 10.3390/pharmaceutics13111902

**Published:** 2021-11-09

**Authors:** Ariane Leite Rozza, Fernando Pereira Beserra, Ana Júlia Vieira, Eduardo Oliveira de Souza, Carlos Alberto Hussni, Emanuel Ricardo Monteiro Martinez, Rafael Henrique Nóbrega, Cláudia Helena Pellizzon

**Affiliations:** 1 Department of Structural and Functional Biology, Institute of Biosciences, São Paulo State University (UNESP), Dr. Antonio Celso W Zanin Street, 250, Botucatu 18618-689, Brazil; Fernando.beserra@unesp.br (F.P.B.); Julia.vieira@unesp.br (A.J.V.); Eduardo_3425@hotmail.com (E.O.d.S.); Erm_martinez@yahoo.com.br (E.R.M.M.); Rafael.nobrega@unesp.br (R.H.N.); Claudia.pellizzon@unesp.br (C.H.P.); 2 Department of Surgery and Veterinary Anesthesiology, School of Veterinary Medicine and Animal Science, São Paulo State University (UNESP), Dr. Walter M Correa Street, Botucatu 18618-689, Brazil; carlos.hussni@unesp.br

**Keywords:** skin wound healing, menthol, inflammation, gene expression, antioxidant, TNF-α, IL-10, GSH

## Abstract

Wound healing involves inflammatory, proliferative, and remodeling phases, in which various cells and chemical intermediates are involved. This study aimed to investigate the skin wound healing potential of menthol, as well as the mechanisms involved in its effect, after 3, 7, or 14 days of treatment, according to the phases of wound healing. Skin wound was performed in the back of Wistar rats, which were topically treated with vehicle cream; collagenase-based cream (1.2 U/g); or menthol-based cream at 0.25%, 0.5%, or 1.0% over 3, 7, or 14 days. Menthol cream at 0.5% accelerated the healing right from the inflammatory phase (3 days) by decreasing mRNA expression of inflammatory cytokines TNF-α and Il-6. At the proliferative phase (7 days), menthol 0.5% increased the activity of antioxidant enzymes SOD, GR, and GPx, as well as the level of GSH, in addition to decreasing the levels of inflammatory cytokines TNF-α, IL-6, and IL-1β and augmenting mRNA expression for Ki-67, a marker of cellular proliferation. At the remodeling phase (14 days), levels of inflammatory cytokines were decreased, and the level of Il-10 and its mRNA expression were increased in the menthol 0.5% group. Menthol presented skin wound healing activity by modulating the antioxidant system of the cells and the inflammatory response, in addition to stimulating epithelialization.

## 1. Introduction

Skin wound healing involves an array of biochemical, molecular, cellular, and tissue processes [1]. The physiological events that lead to wound repair can be divided into three dynamic phases: inflammatory, proliferative, and tissue remodeling. These overlapping steps encompass a network of cellular communication that ensures the progress of the healing process [2].

Hemostasis and inflammation occur immediately after the tissue damage. Hemostasis is assisted by the formation of a fibrin hemostatic plug, preventing the bleeding. The prevention of infection is a process orchestrated by the inflammatory cells. Neutrophils and macrophages make phagocytosis of pathogens and tissue debris, as well as secret growth factors and cytokines, essential signaling molecules for triggering the proliferative phase [3,4].

The development of granulation tissue is the peculiarity of the proliferative phase. The proliferation of epithelial cells constitutes the new epidermis in a process named epithelialization. The contraction of myofibroblasts approximates the wound edges, reducing the wound area. Many kinds of cytokines and growth factors play an essential role in skin wound repair [5].

The last stage of wound repair is the extracellular matrix remodeling, in which the apoptosis of macrophages and myofibroblasts occur, ending the formation of the granulation tissue. The type III collagen is replaced with type I collagen in a process that can last for months [6].

The treatment of skin wounds is responsible for the largest investment in research and development among all skin diseases [7] and is among the most critical issues in medical science [5]. Clinically, several drugs have been developed, aiming to accelerate the wound healing process but presenting side effects or low efficiency. In modern medical sciences, searching for drugs that accelerate wound healing with minimal pain, discomfort, and scarring is a promising area of research [6]. Several studies have successfully tested the effects of medicinal plants in skin wound healing, in which the extracts or their isolated compounds accelerated skin wound closure, inhibited local inflammation, and increased cellular antioxidant defense [8,9]. Plant-based therapy is known to accelerate wound healing as well as maintaining the aesthetics in a natural way [10]; this is the reason why more than 70% of wound healing pharmaceutical products are derived from plants [11].

Menthol is a monocyclic monoterpene found in the essential oil of some species of the genus *Mentha* [12]. Menthol is an agonist of transient receptor potential melastatin-8 channels, cold receptors in the sensory nerves of the skin. For this reason, menthol exerts a cooling sensation when applied to the skin and mucosal membranes [13]. The activation of TRPM8 channels does not influence skin wound healing [14]. Menthol also activates TRPV3 channels and presents a bimodal effect in TRPA1 and TRPV1 [15]. Some studies have shown that the activation of TRPV3 channels contributes to wound healing in the skin [16], oral mucosa [17], and corneal epithelial cells [18]. Furthermore, the inhibition of TRPA1 and TRPV1 channels is involved with the pain-relieving effect of menthol [19]. Menthol is widely used in cosmetics, as well as in the medicinal preparations for the relief of pain [20] and respiratory conditions [21].

Previous studies have shown that menthol has antinociceptive [22], antimicrobial [23], and antiulcerogenic properties [24,25]. We hypothesized that these biological effects could make menthol a promising molecule to be used in wound healing, and in this study, we aimed to investigate the potential of menthol in skin wound healing in Wistar rats, as well as to assess biochemical and molecular parameters involved in the effect, at the three phases of wound healing.

## 2. Material and Methods

### 2.1. Animals

Male Wistar rats (200–250 g, 8 to 10 weeks old) were acquired from Central Animal House of UNESP Botucatu. The rats were housed in cages for acclimatization with temperature (21 ± 2 °C) and humidity (60 ± 1%) controlled with 12 h light/dark cycles. The rats were fed a certified diet. All efforts were made to avoid animal suffering. The researchers followed the guidelines of the Canadian Council on Animal Care. The experimental protocols were approved by the Institutional Animal Care and Use Committee (protocol 793-CEUA).

### 2.2. Development of Creams Used in Skin Wound Treatment

(−)-Menthol (<99% purity) was purchased from Merck and incorporated into Lanette cream at concentrations of 0.25% (ME0.25), 0.5% (ME0.5), and 1% (ME1.0). Collagenase was incorporated into Lanette cream and used as a reference drug (1.2 U/g). Lanette cream alone was used as the vehicle group.

### 2.3. Skin Wound Procedure and Treatment

At first, the rats were anesthetized using an intraperitoneal injection of ketamine (80 mg/kg) and xylazine (10 mg/kg), before a single subcutaneous injection of ketoprofen (100 mg/kg) to alleviate postoperative discomfort. The back of the rats was previously trichotomized, and a skin wound was performed using a punch with 2 cm of diameter. After the wounding procedure, the rats were moved to individual cages and randomly distributed into six groups of treatment (*n* = 8): vehicle (Lanette cream), collagenase-based cream (1.2 U/g), or three crescent concentrations of menthol-based cream (ME.025, ME0.5, or ME1.0). Once a day, the wound region was measured by planimetry and photographed using a ruler as a scale, and the creams were applied in the skin wounds. The rats were treated over 3, 7, or 14 days, according to the phases of wound healing (inflammatory, proliferative, and tissue remodeling phase) [26]. At the end of each period, the rats were euthanized by deepening the anesthetic plan. The wound region was collected to perform histological, biochemical, and molecular assays; livers were also collected to perform antioxidant assays. The samples were preserved at −80 °C or destined to histological analysis.

### 2.4. Wound Area Contraction Rate

The wound area was calculated using Adobe Photoshop CS3, according to the following equation: (wound area at day one—wound area at the day 3, 7 or 14/wound area at day 1) × 100. The result was expressed as the percentage of wound contraction [27].

### 2.5. Histological Analysis

Skin samples were fixed, embedded in paraffin, cut to a thickness of 5 µm, and stained with hematoxylin and eosin (HE) for histological evaluation. Photomicrographs were taken using a camera attached to the microscope.

### 2.6. Analysis of Antioxidant and Myeloperoxidase (MPO) Activities

These assays were performed in the liver [28]. The superoxide dismutase (SOD), glutathione peroxidase (GPx), and glutathione reductase (GR) activities were evaluated according to Winterbourn et al. [29], Yoshikawa et al. [30], and Carlberg and Mannervick [31], respectively. The glutathione (GSH) levels were measured [32]. MPO activity was measured to assess the neutrophils activation [33].

### 2.7. Analysis of Anti-Inflammatory Activity

Skin samples were diluted in phosphate buffered saline and centrifuged at 4 °C; then, the level of cytokines was measured using enzyme-linked immunosorbent assay (ELISA) kits for interleukin-1β (IL-1β), interleukin-6 (IL-6), tumor necrosis factor-α (TNF-α), and interleukin-10 (IL-10) from R&D Systems, following the manufacturer’s instructions.

### 2.8. Analysis by Real-Time Quantitative Gene Expression: RT-qPCR

The total skin RNA was extracted and treated to avoid DNA contamination, and subsequently, cDNA synthesis was performed [34]. For qPCR, Cq values were determined using a SYBR Green kit (Invitrogen, Carlsbad, CA, USA), the gene Ef-1α was used as a reference, and subsequently the values were calibrated with ddCT. qPCR reactions (20 µL) used 900 nM for each primer and 700 ng of total RNA. The reactions were performed in duplicate, according to the manufacturer’s protocol, and relative gene expression profiles were calculated [35]. qPCR was conducted using designed and specific primers for *Rattus norvegicus* (Table 1).

### 2.9. Statistical Analysis

The results were analyzed using one-way ANOVA followed by Dunnett’s test. The results were presented as the mean ± s.e.m., and statistical significance was set at *p* < 0.05. All analyses were performed using Graph Pad Prism.

## 3. Results

### 3.1. Skin Wounds Healing

**Figure 1:**
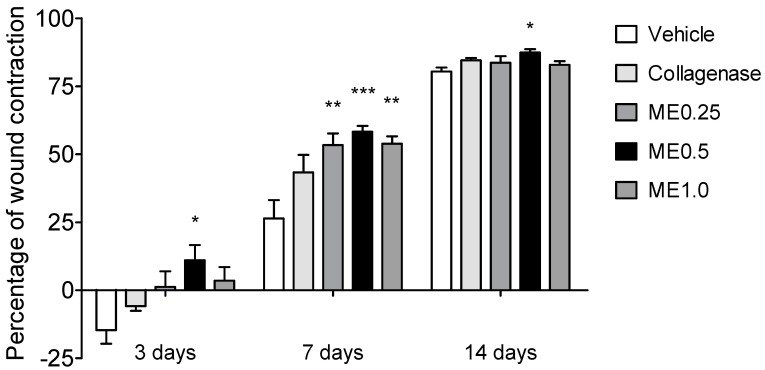
Percentage of skin wound contraction at the back of rats after 3, 7, or 14 days of treatment with vehicle, collagenase cream, ME.025, ME0.5, or ME1.0. ANOVA, Dunnett’s test; * *p* < 0.05, ** *p* < 0.01, *** *p* < 0.001.

We used an excisional wound model for investigating the influence of menthol on the healing process in rats. The sequence of wound healing can be assessed by daily monitoring of the wound contraction [36]. At day 3, only the rats treated with ME0.5 presented a significant percentage of wound contraction (11%, *p* < 0.05) in comparison to the vehicle group. Rats treated with ME0.25, ME1.0, or collagenase cream presented similar wound contraction when compared to the vehicle group (Figure 1). At the end of 7 days of treatment, all groups treated with menthol presented higher percentage of wound contraction in comparison to the vehicle group. Respectively, the percentage of wound closure was 53%, 58%, and 54% for ME0.25, ME0.5 and ME1.0 (*p* < 0.05, *p* < 0.01, and *p* < 0.05; Figure 1). Finally, after 14 days of treatment, the group treated with ME0.5 maintained a higher percentage of wound closure (87%, *p* < 0.05), indicating that the healing process in this group was accelerated during all the treatment period. The other groups presented wound contraction percentage similar to the vehicle group (Figure 1). Since menthol-based cream at the concentration of 0.5% was the effective concentration in the three periods tested, we used the samples of this group for all subsequent analyses.

**Figure 2:**
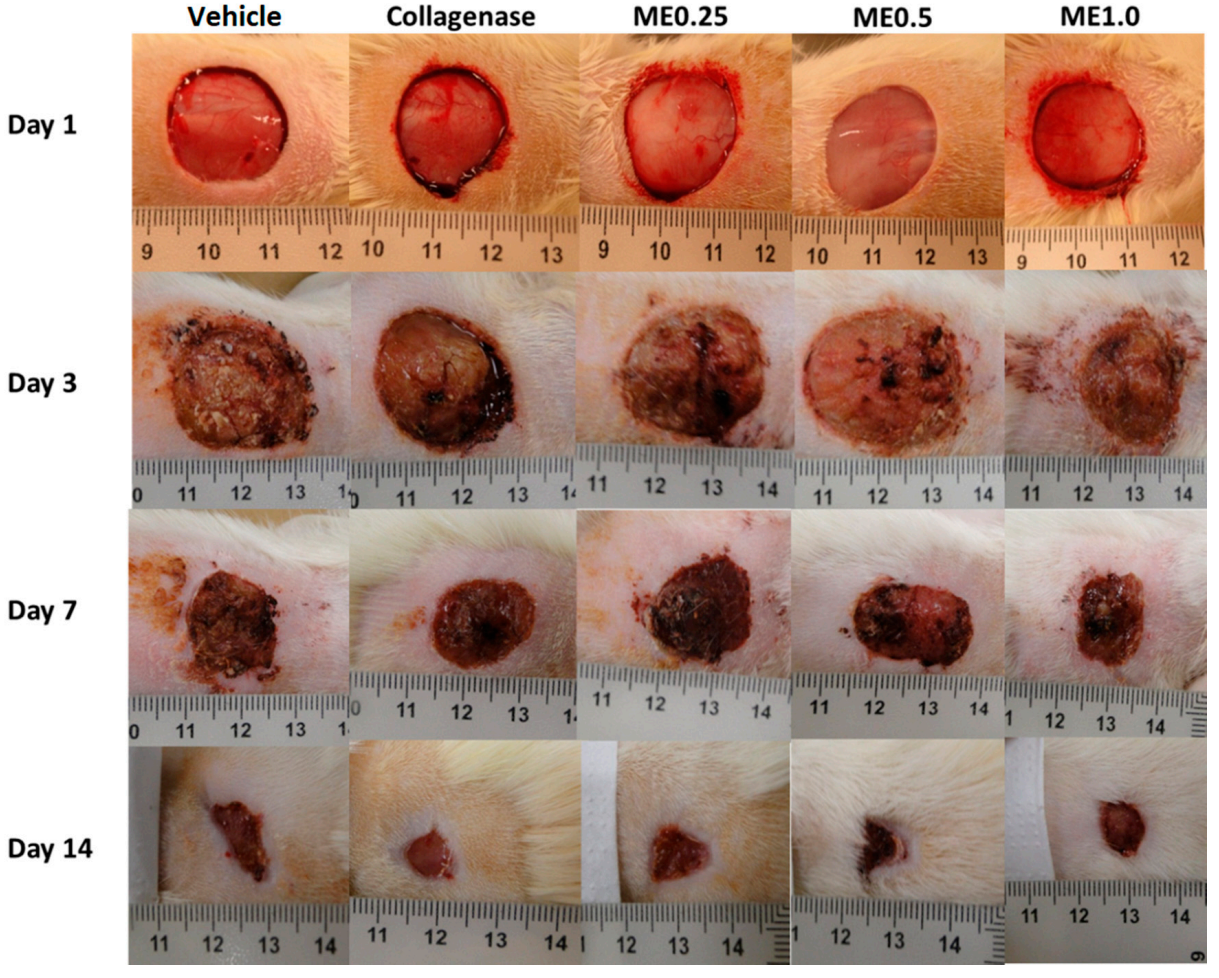
Skin wounds at the moment they were performed and after 3, 7, or 14 days of treatment with vehicle, collagenase cream, ME.025, ME0.5, or ME1.0.

Regarding the appearance of the wounds, in the first three days, we observed redness, inflammation, and swelling in all groups, which could explain the decrease in wound contraction (except for ME0.5) observed in Figure 1. Such characteristics were gradually disappearing and giving rise to the granulation tissue, indicating the onset of the proliferative phase. This process can be seen in Figure 2.

### 3.2. Histological Analysis

**Figure 3:**
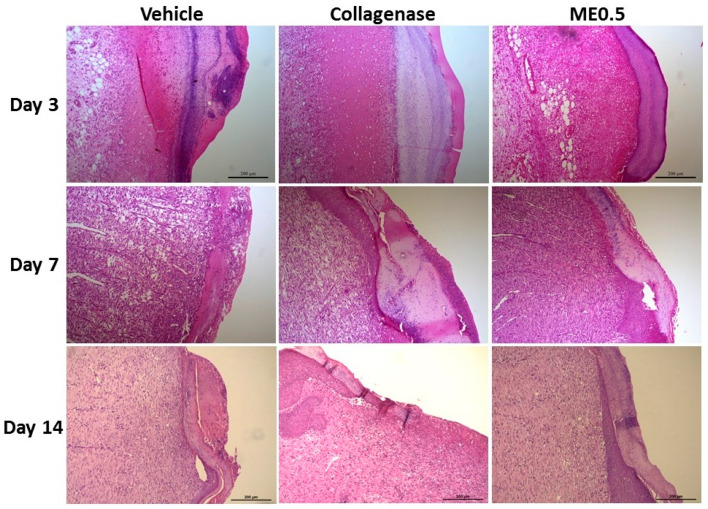
Representative histological sections of skin wounds after 3, 7, or 14 days of treatment with vehicle, collagenase cream, or ME0.5 (HE staining). Scale bar: 200 µm.

At day 3, there was an intense inflammatory infiltrate in the wound region and in the dermis. At day 7, the granulation tissue formed in the menthol-treated group recovered a normal epithelium, which could be seen in the margin of the wound, an effect that was more pronounced after 14 days of treatment, when the reestablishment of the epidermis on the wound margins occurred, growing towards the center. In the dermis, there were cells of the connective tissue (mainly fibroblasts) and deposition of collagen fibers, demonstrating effective extracellular matrix remodeling (Figure 3).

### 3.3. MPO and Antioxidant Activities

SOD, GPx, and GR activities were measured, in addition to the quantity of GSH. Such enzymes and protein scavengers free radicals and prevent oxidative damage. The effectiveness of the wound healing process is directly related to the antioxidant ability of the therapeutic agent, since removing free radicals significantly accelerates the healing process, reducing tissue damage and wound infection [9,37]. The activity of MPO was also measured as an indicator of pro-oxidant and pro-inflammatory activity. After three days of treatment, ME0.5 was not able to induce alterations in the activity or quantity of the measured parameters. However, at day 7, all antioxidative parameters were increased (activities of GR, GPx, and SOD, and the level of GSH), and the activity of MPO was not altered. After 14 days of treatment, the antioxidative parameters were normalized, and the activity of MPO was decreased (Table 2).

### 3.4. Anti-Inflammatory Activity

Cytokines are messenger molecules of the immune system that perform the interaction between immune, nervous, and endocrine systems; several cytokines are involved in the skin wound healing process [38]. After three days of treatment, the levels of cytokines were not different among groups. At day 7, the treatment with ME0.5 was able to decrease inflammatory cytokine production, which persisted until day 14, when the levels of IL-10 were increased (Table 3).

### 3.5. Gene Expression

**Figure 4:**
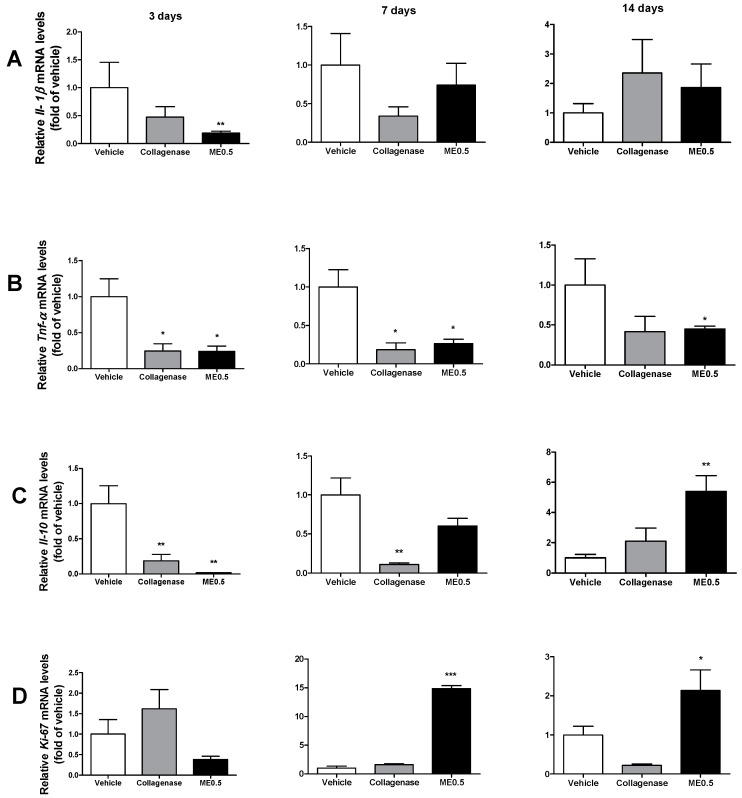
Levels of Il-1β (**A**), TNF-α (**B**), IL-10 (**C**), and Ki-67 (**D**) mRNA expression in skin wounds after 3, 7, or 14 days of treatment with vehicle, collagenase cream, or ME0.5. ANOVA, Dunnett’s test; * *p* < 0.05, ** *p* < 0.01, *** *p* < 0.001.

At day 3, mRNA expression for Il-1β, TNF-α, and Il-10 was decreased in the ME0.5 group (Figure 4A–C). After seven days of treatment, the mRNA expression of TNF-α was decreased, which persisted until day 14 (Figure 4B), when mRNA expression of Il-10 was increased (Figure 4C). After 7 and 14 days of treatment, the mRNA expression of Ki-67 was augmented in this group (Figure 4D), which could indicate cell proliferation activity to recover the wounded area.

## 4. Discussion

Modaressi et al. [39] showed that the topical application of *Mentha piperita* ointment at 4% and 8% accelerated the healing of infected skin wounds in mice after 4, 8, 12, and 16 days of treatment. The authors attributed the effect mainly to the modulation of the mRNA expression of the inflammation-related genes by menthol. Babamohamadi et al. [40] evidenced that a *Mentha piperita* gel (applied three times a day up to 14 days) prevented the development of pressure ulcers in hospitalized patients. In this study, we intended to prospect the effect of menthol in skin wound healing. Skin wounds were developed in rats and treated with menthol-based creams for 3, 7, or 14 days, according to previously described stages of wound healing: inflammatory, proliferative, and tissue remodeling phase [9]. This is the first study evaluating the in vivo wound healing potential of menthol, and we have demonstrated that, topically applied, menthol at the concentration of 0.5% accelerated the wound closure in 3, 7, and 14 days of treatment. The mechanisms potentially involved in the effect are discussed as follows.

Several studies have demonstrated that, at the site of skin wounds, the concentration of reactive oxygen species (ROS) increases and antioxidant production decreases, leading to a delayed wound healing [41]. Therefore, compounds with antioxidant potential are good therapeutic agents for accelerating the wound healing process [42]. In the inflammatory phase, neutrophils and macrophages produce large amounts of ROS, which directly attack pathogens but also damage the healthy surrounding tissues. Superoxide anions are dismutated to H_2_O_2_ and oxygen by SOD. Subsequently, H_2_O_2_ is detoxified by peroxidases such as GPX, preventing the generation of hydroxyl radicals, the most harmful forms of ROS, requiring GSH as an electron donor. Such antioxidant enzymes are abundantly present in the skin and are crucial to scavenging ROS during wound healing [43]. GSH is a non-enzymatic antioxidant converted to inactive form after reacting with ROS. Its levels are decreased in cutaneous wounds in comparison to healthy skin. The inhibition of ROS production triggers angiogenesis and fibroblast proliferation, stimulating the skin wound closure. MPO is an oxidant and inflammatory enzyme released by neutrophils. Excessive neutrophils in the wound site trigger excessive ROS production by MPO, delaying the wound healing [44]. We demonstrated that, in the inflammatory phase, topical treatment with menthol 0.5% did not alter the systemic activities of GR, GPx, SOD, and MPO, nor the level of GSH. However, in the proliferative phase, menthol increased the activities of GR, GPx, and SOD, as well as the level of GSH, evidencing an antioxidant effect at this phase. At the tissue remodeling phase, menthol decreased MPO activity; the enzyme activity and GSH level were normalized. We hypothesized that the cells produced at the proliferative phase helped to increase the production of antioxidant molecules in an effect that was normalized after 14 days of treatment (tissue remodeling phase).

During wound healing, monocytes arrive at the wound site and turn into macrophages, releasing several inflammatory mediators and cytokines, and producing ROS. TNF-α, IL-1β, and IL-6 are inflammatory cytokines that play significant roles in the inflammatory process during skin wound healing because they coordinate actions of different types of cells [38]. Secreted cytokines induce chemokines of chemotaxis, attracting immune cell to the site of the injury, contributing to skin repair and homeostasis [45]. We did not find alterations in cytokine levels induced by menthol after 3 days of treatment, suggesting that the treatment did not inhibit the inflammatory phase. However, at proliferative and extracellular matrix remodeling phases, menthol decreased the levels of TNF-α, IL-6, and IL-1β. In addition, at the remodeling phase, menthol treatment increased the activity of IL-10, which dampens the expression of pro-inflammatory cytokines, as well as anti-scarring function [3]. Our results are in accordance with several studies reported in the literature. Such studies showed that the inflammatory phase restores homeostasis, and that, in the proliferative phase, fibroblasts and other cells from the connective tissue infiltrate the wound site and secrete cytokines, attracting keratinocytes and leading to re-epithelialization [26,46]. Preventing prolonged inflammation by suppressing the production of inflammatory cytokines is a desirable target for wound healing products; excessive inflammation results in chronic wounds and scar formation [47]. The modulation of cytokines production by menthol denotes an anti-inflammatory activity, which is confirmed in the literature using different animal models [48,49].

Epithelialization has a key role in wound healing, especially at the proliferative phase, and is used as an index of its success [50]. We observed a variable correlation between the relative mRNA expression and the levels of cytokines obtained by ELISA method, which leads us to suppose that menthol acts through a post-transcriptional or/and post-translational regulation; however, it also can be credited by differences in mRNA and proteins half-lives [51]. The mRNA expression levels of TNF-α and Il-10 in the vehicle group are in accordance with the results obtained by Kubo et al. [52], who investigated the temporal expression of wound healing-related genes after skin injury. Tatiya-Aphiradee et al. [53], Luo et al. [54], and Ram et al. [55] also found similar results for the mRNA expression level of TNF-α after the treatment of skin wounds with natural products. The increased mRNA expression for Ki-67 at the proliferative phase in menthol-treated rats indicates enhanced epithelialization, resulting in a better quality of the granulation tissue and the diminishing of the wound surface [56].

## 5. Conclusions

We reported for the first time that menthol accelerates skin wound healing through several mechanisms. At the inflammatory phase, menthol decreased mRNA expression of inflammatory cytokines. At the proliferative phase, menthol displayed antioxidant and anti-inflammatory activities, in addition to stimulating cell proliferation, contributing to the formation of granulation tissue. At the last phase (tissue remodeling), menthol reinforced its anti-inflammatory activity by increasing the mRNA expression and the production of IL-10, as well as decreasing the levels of inflammatory cytokines and MPO activity. To our knowledge, this is the first study about skin wound healing activity from menthol, and these innovative findings could enable this compound as a potential substance to be used in skin wound healing.

## Figures and Tables

**Table 1:** Specifications of primers used in RT-qPCR.

Gene	Size (bp)	Sequence 5′–3′	MT	NCBI Reference Sequence
*Il-1β*	93	FW: AGGCTTCCTTGTGCAAGTGT	60 °C	NM_031512.2
		RV: AGGTCATTCTCCTCACTGTCG		
*Il-6*	92	FW: TCATTCTGTCTCGAGCCCAC	60 °C	NM_012589.2
		RV: CTCCGCAAGAGACTTCCAGC		
*Il-10*	95	FW: GACGCTGTCATCGATTTCTCC	60 °C	NM_012854.2
		RV: GCTCCAAGACAAAGGTGTCTAC		
*Tnf-α*	100	FW: ATGGGCTCCCTCTCATCAGT	60 °C	NM_012675.3
		RV: TGGTTTGCTACGACGTGGG		
*Ki-67*	100	FW: GGGTTTCCAGACACCAGACC	60 °C	NM_001271366.1
		RV: CCAGGAAGACCAGTTAGAACC		
*Ef-1α*	91	FW: CTTTGGACTGCATTCTGCCG	60 °C	NM_175838.1
		RV: GTGCCAATGCCGCCAATTTT		

Bp: base pairs, MT: melting temperature, FW: forward, RV: reverse.

**Table 2:** Quantity of GSH and activities of GR, GPx, SOD, and MPO after 3, 7 or 14 days of wound treatment with vehicle, collagenase cream or ME0.5.

Period	Treatment	GSH	GR	GPx	SOD	MPO
3 days	Vehicle	3.29 ± 0.28	57.61 ± 2.89	26.50 ± 4.17	33.21 ± 2.07	0.23 ± 0.03
	Collagenase	3.72 ± 0.46	60.12 ± 3.52	22.99 ± 3.20	31.17 ± 1.87	0.35 ± 0.05
	ME0.5	2.46 ± 0.22	58.20 ± 0.88	24.27 ± 2.87	32.13 ± 3.74	0.39 ± 0.01
7 days	Vehicle	3.43 ± 0.35	31.93 ± 4.14	19.10 ± 3.03	36.42 ± 2.63	0.12 ± 0.01
	Collagenase	6.01 ± 0.93	34.28 ± 3.66	21.76 ± 2.89	41.63 ± 4.32	0.10 ± 0.00
	ME0.5	11.39 ± 0.92 ***	48.83 ± 4.12 *	31.73 ± 2.27 *	50.86 ± 5.82 *	0.11 ± 0.01
14 days	Vehicle	13.65 ± 2.18	96.34 ± 4.01	62.88 ± 3.57	31.31 ± 2.54	0.47 ± 0.09
	Collagenase	12.03 ± 0.75	97.00 ± 4.55	58.20 ± 2.86	34.48 ± 1.46	0.30 ± 0.02
	ME0.5	10.82 ± 1.00	82.89 ± 2.57	53.31 ± 1.80	27.35 ± 0.45	0.24 ± 0.02 *

GSH level: nmol/mg of protein; GPx and GR: nmol/min/mg of protein; SOD and MPO: U/mg of protein. ANOVA, Dunnett’s test, * *p* < 0.05; *** *p* < 0.001.

**Table 3:** Levels of IL-1β, IL-6, TNF-α, and IL-10 in skin wounds after 3, 7, or 14 days of treatment with vehicle, collagenase cream, or ME0.5.

Period	Treatment	IL-1β	IL-6	TNF-α	IL-10
3 days	Vehicle	1238.00 ± 137.90	2083.00 ± 219.00	205.50 ± 34.11	203.60 ± 66.43
	Collagenase	1176.00 ± 186.60	1730.00 ± 264.30	202.70 ± 64.39	181.70 ± 51.34
	ME0.5	1177.00 ± 226.50	1505.00 ± 308.10	120.10 ± 27.55	397.60 ± 158.90
7 days	Vehicle	807.20 ± 165.80	1893.00 ± 566.80	80.35 ± 27.41	144.90 ± 35.17
	Collagenase	579.50 ± 104.80	820.80 ± 218.70 *	45.33 ± 13.75	457.50 ± 149.70
	ME0.5	266.60 ± 68.05 *	570.10 ± 98.06 **	21.19 ± 4.03 *	262.30 ± 78.13
14 days	Vehicle	2230.00 ± 254.70	1653.00 ± 177.30	138.30 ± 40.96	217.10 ± 70.60
	Collagenase	1369.00 ± 207.70 *	1113.00 ± 149.00	30.52 ± 8.73 **	645.50 ± 156.80
	ME0.5	937.40 ± 142.20 ***	831.70 ± 173.30 **	31.58 ± 10.02 **	1128.00 ± 161.80 **

The results were expressed as pg/mg of protein. ANOVA, Dunnett’s test; * *p* < 0.05, ** *p* < 0.01, *** *p* < 0.001.

## Data Availability

Data will be provided upon request.
